# Design of Enhanced Rotation Locked Loop for Roll Angle Estimation of Rotating Vehicle in a Weak GPS Signal Environment

**DOI:** 10.3390/s19010080

**Published:** 2018-12-26

**Authors:** Hun Cheol Im, Deok Won Lim, Sang Jeong Lee

**Affiliations:** 1 Agency for Defense Development, Daejeon 34186, Korea; peterim@add.re.kr; 2 Satellite Navigation Team, Korea Aerospace Research Institute, Daejeon 34133, Korea; dwlim@kari.re.kr; 3 Department of Electronics Engineering, Chungnam National University, Daejeon 34134, Korea

**Keywords:** GPS, rotating vehicle, roll angle estimation, RLL

## Abstract

In order to estimate the roll angle of a rotating vehicle, an enhanced rotation locked loop (RLL) algorithm is proposed in this paper. The RLL algorithm estimates the roll angle by using the property that the power of the GPS signal measured at the receiver of a rotating vehicle changes periodically. However, in case the received GPS power is decreased, the performance of the conventional RLL algorithm degrades, or it cannot estimate the roll angle anymore, therefore, for operating the RLL algorithm in a weak signal environment, this paper designs a method to increase the signal-to-noise ratio (SNR) by overlapping multiple GPS signals’ correlator outputs and a method to compensate the decreased response of a rotation discriminator at low-signal strength. Through computer simulations, the performance of the proposed algorithm is verified and it is shown that the roll angle can be estimated stably even at a weak signal environment down to 29 dB–Hz of C/N0.

## 1. Introduction

The global positioning system (GPS), which provides accurate position, velocity, and time all over the world, has been widely used in a military and civilian areas [1]. In recent years, research on using GPS signals in a rotating vehicle have been carried out steadily. In particular, studies have been conducted to estimate the roll angle and integrate it with the navigation system of a rotating vehicle such as an artillery shell which moves with predictable trajectory [2,3,4,5,6,7,8,9].

Doty, et al. [2,3,4] proposed a GPS roll angle determination algorithm for estimating the roll angle in a rotating vehicle and utilized it to integrate with the accelerometer output to perform navigation. Kim, et al. [5] proposed a method to compensate the carrier tracking loop by estimating the roll angle using XOR (exclusive OR) logic and calculating the carrier frequency in a rotating vehicle. Park et al. [6]. proposed a roll angle estimation algorithm using extended Kalman filter with pitch and roll rate measurements and verified the performance for estimating the roll angle by simulations and experiments for the vehicle with a rotating frequency of 1–1.5 Hz. However, this method needs two rate gyroscopes, and the convergence time is rather large since the Kalman filter should estimates all the state variables. Moreover, this method has a limitation that it can be used only for the constant rotation frequency. Choi et al. [7] proposed a rotation locked loop (RLL) algorithm to estimate the roll angle and performed detailed analysis of that algorithm under various conditions which do not include weak signal environments. Im et al. [8,9] proposed an algorithm for estimating the roll angle using the RLL algorithm in a multi-antenna mounted vehicle and compensating the carrier tracking loop using the carrier frequency obtained in the roll angle estimation algorithm. However, because these methods did not consider the situation where the strength of the satellite signal measured at the receiver becomes low, their performances degrade or they do not even work at weak signal environments.

Therefore, this paper focuses on estimating the roll angle of a rotating vehicle in a weak signal environment. For this purpose, this paper proposes an enhanced RLL algorithm, which increases the signal-to-noise ratio (SNR) by overlapping multiple GPS signals’ correlator output and compensates the decreased response of a rotation discriminator in a weak signal environment.

This paper is organized as follows. The proposed algorithm is described in Section 2, the performance of the proposed algorithm is verified by computer simulations in Section 3. Finally, the conclusions are given in Section 4.

## 2. Enhanced RLL Algorithm for a Weak GPS Signal Environment 

### 2.1. Signal Overlapping Algorithm

This section presents a method to increase the SNR by overlapping correlator outputs of multiple GPS signals in a weak signal environment. A GPS receiver acquires multiple GPS signals simultaneously. Therefore, by synchronizing and overlapping the received signals, the SNR can be increased. The proposed algorithm is based on the property that if N signals with uncorrelated noises of normal distribution are added each other, the power of the signal increases by $N^{2}$ times and the power of the noise increases by N times. As a result, the SNR of the overlapped signal increases by N times.

**Figure 1:**
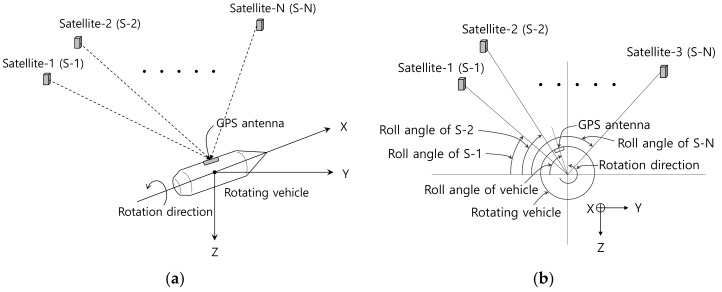
The geometry of rotating vehicle and satellites: (**a**) 3-dimensional view; (**b**) 2-dimensional view (Rear view).

In this paper, the geometry of the rotating vehicle and satellites considered in this paper are shown in Figure 1. The rotating vehicle is assumed to have one GPS antenna on the surface of its skin, and the roll angle of the vehicle is defined as the angle between –Y axis and the vehicle’s center to GPS antenna line as shown in Figure 1b. The roll angle of each satellite is defined as the angle between –Y axis and the vehicle to satellite line of sight angle.

Assuming that the vehicle’s position and attitude (pitch and heading) are known, the azimuth and elevation angle of the satellite relative to the vehicle can be obtained, then the roll angle of the satellite can be acquired based on the pitch and heading angle of the vehicle. After then the time delays to synchronize all the received satellite signals can be obtained using the rotation frequency of the vehicle. The pseudo code to obtain the time delay is described in Table 1. In case of rotating artillery shell, accurate initial position and attitude can be transferred from the fire control system and it moves with predictable trajectory after fire. Therefore, estimated pitch and heading angle can be easily obtained during flight. Here, the rotation frequency of the vehicle (f) is used to compute the time delay (t_d_) to synchronize multiple satellite signals. However, since the true rotation frequency is not known, the estimated value ($\hat{f}$) can be used. Therefore, initial rotation frequency is required to use the overlapping algorithm and the scheme for acquiring initial rotation frequency was proposed in [7]. In this paper, we focused on the tracking performance of the proposed RLL algorithm when the rotation frequency changes. Therefore, initial roll angle and rotation frequency for convergence of the RLL tracking loop were assumed to be known a priori. Signal overlapping algorithm is insensitive to the rotation frequency error and a low-precision low-cost gyroscope can be used if necessary. After the RLL algorithm is converged, it can estimate rotation frequency as well as roll angle, therefore this output can be used to compute the time delay for the overlapping algorithm.

**Figure 2:**
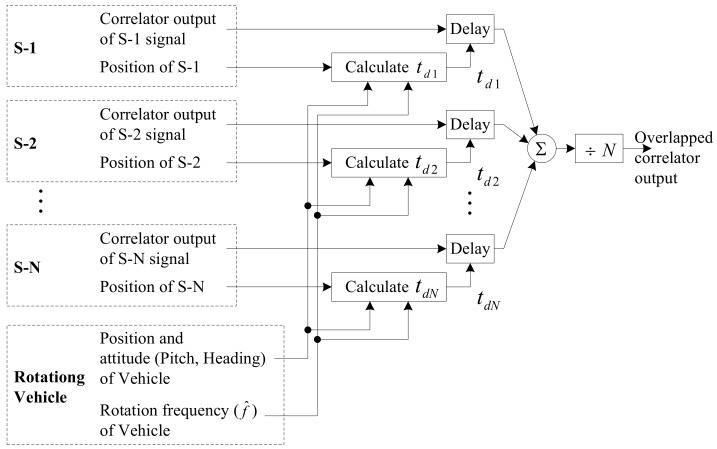
Block diagram of signal overlapping algorithm.

**Figure 3:**
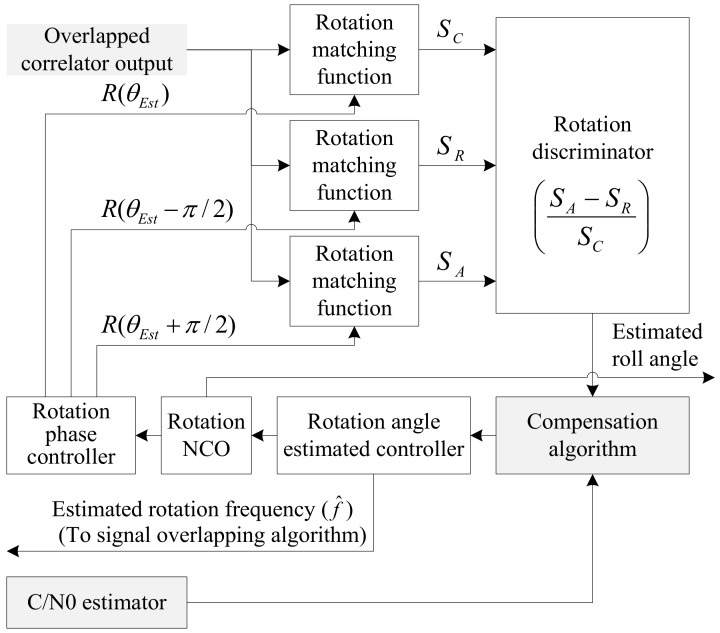
Enhanced rotation locked loop (RLL) algorithm with overlapped signal.

The block diagram of the overlapping algorithm is shown in Figure 2. First, time delays ($t_{d1},t_{d2},\ldots ,t_{\mathrm{dN}}$) are computed as described above to synchronize the received GPS signals using the positions of the satellites and the position, attitude, and the rotation frequency of a vehicle. Next, correlator outputs of all satellites are delayed for each time delay and summed all. Finally, the summed correlator outputs are divided by the number of signals, which becomes the overlapped correlator output. After the RLL algorithm is converged, the output of the RLL algorithm is used as the estimated rotation frequency (See Figure 3).

Proposed RLL algorithm is shown in Figure 3, which is modified from conventional RLL algorithm to use overlapped correlator output as its input. Rotation phase controller generates 90 degree retarded and advanced phases relative to the currently estimated roll angle. Each three rotation matching function extract centered, retarded, and advanced component from overlapped correlator output according to the different phase input, where the three components have similar functions to the prompt, late, and early replica code in delay lock loop (DLL) respectively. Rotation discriminator generates angle error (E) between the true and estimated roll angle, where E = (S_A_ − S_R_)/S_C_. Rotation angle estimated controller and rotation NCO (numerically controlled oscillator) generate control input using the angle error, which has the second order controller structure. Rotation angle estimated controller generates estimated rotation frequency and it is used for the rotation frequency in the overlapping algorithm. The structure of RLL can be compared to DLL where (a) rotation phase controller, (b) rotation matching function, (c) rotation discriminator, (d) rotation angle estimated controller, (e) rotation NCO is similar to (a’) 2-bit shift register, (b’) mixer and integrate and dump, (c’) code loop discriminator, (d’) code loop filter, (e’) numerically controlled oscillator, respectively [1]. For more detailed explanations on RLL algorithm refer to [1,7].

### 2.2. Compensation Algorithm

**Figure 4:**
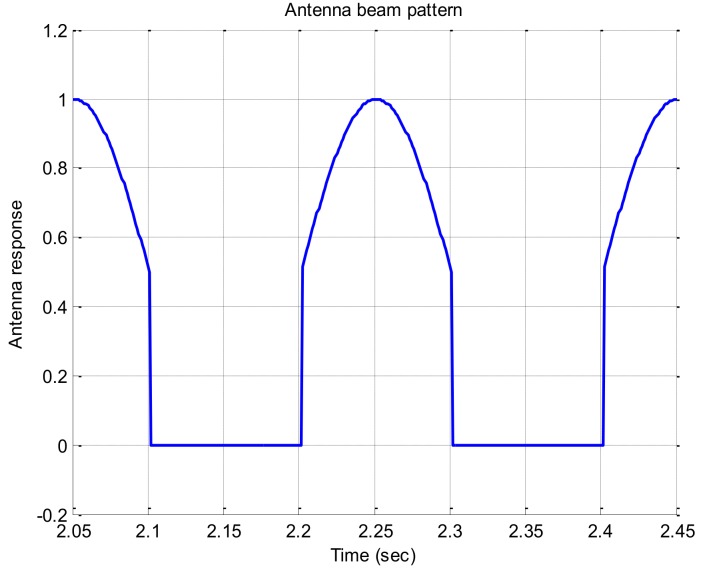
Antenna response for two turn.

**Figure 5:**
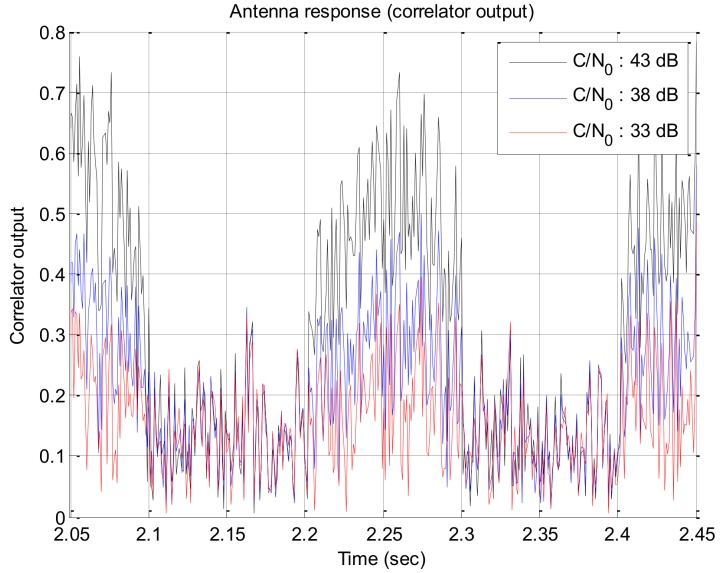
Correlator outputs w.r.t. the signal strength.

**Figure 6:**
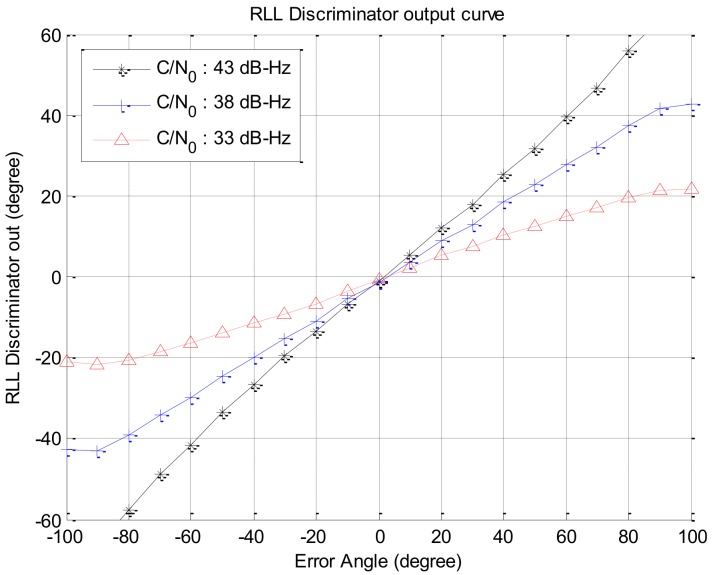
Rotation discriminator outputs w.r.t. the error angles.

**Figure 7:**
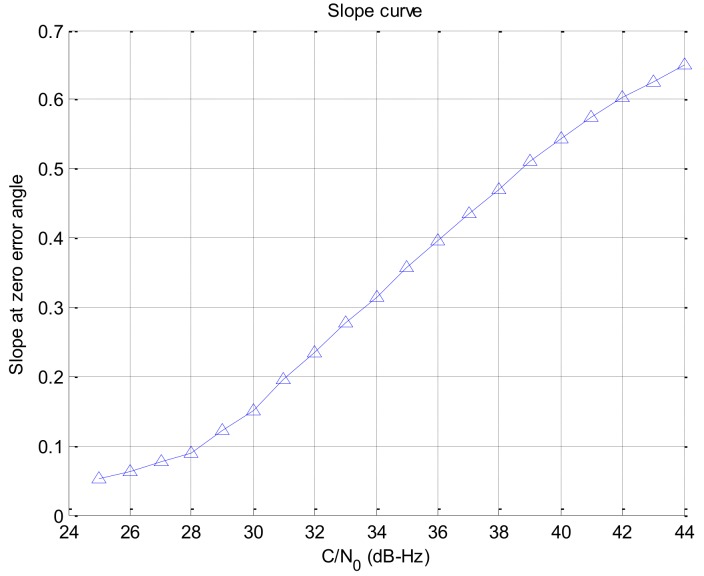
Slope of the rotation discriminator at zero error angle w.r.t the C/N0.

If the signal strength decreases, the response of the rotation discriminator which uses the correlator output decreases too. In this paper, we assumed that an antenna has a beam pattern with 180 degree of beam width as shown in Figure 4. The correlator outputs according to signal strengths are given in Figure 5. The discriminator outputs according to the angle error are shown in Figure 6, and the slopes at the zero angle error according to the signal strength are shown in Figure 7. As shown in Figure 5, Figure 6 and Figure 7, it can be found that the response of the discriminator decreases as the signal strength decreases. If the RLL control gain is designed at the condition where the signal strength is sufficiently high, then the roll angle cannot be estimated quickly enough when the rotation frequency changes, as a result it becomes easy to lose the tracking. Therefore, the response reduced by weak signal strength should be compensated. In order to compensate the response according to the signal strength, in this paper, a look-up table is adopted and the inverse of the slopes according to the signal strength as shown in Figure 7 are stored in the table. These blocks are shown as C/N0 estimator and compensation algorithm in Figure 3.

In addition, the effect of the C/N0 estimation error on the compensation of the discriminator is analyzed. The gain curve of the discriminator could be modeled as the first order linear function, which is described as follows:

(1) y =0.0325 (x−24),

where, x is the C/N0 and y is the slope at zero error angle.

Because the compensation algorithm in Figure 3 uses the inverse of the gain curve in Figure 7 as the compensation value, the control gain (k) for the compensation is as follows:

(2) $$k\;=\;\frac{30.8}{x-24}.$$

If $x_{e}$ is the estimated C/N0 error, the control gain with this error ($k_{e}$) is as follows:

(3) $$k_{e}\;=\;\frac{30.8}{x+x_{e}-24}.$$

The compensation error ($e_{k}$) can be obtained as follows:

(4) $$e_{k}\;=\;\frac{k_{e}-k}{k}\times 100\left(\%\right).$$

**Figure 8:**
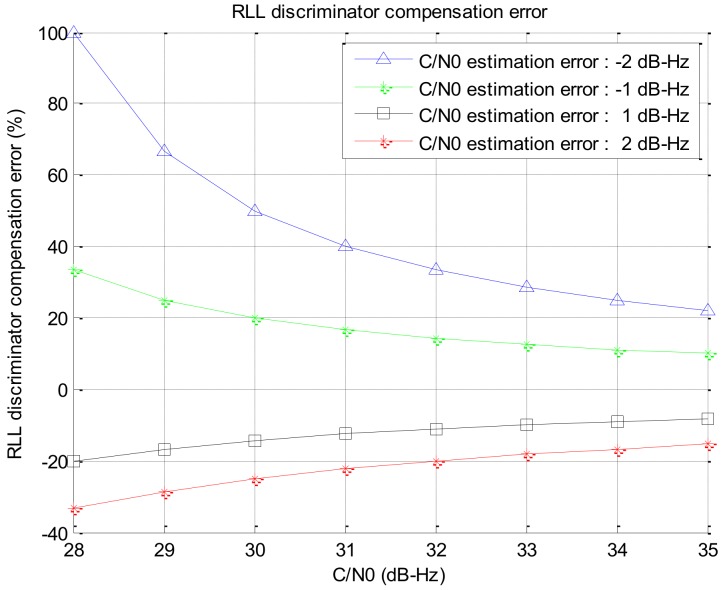
Compensation error w.r.t the estimated C/N0 error.

If the C/N0 estimation error is assumed to be ±1 dB–Hz, ±2 dB–Hz at the C/N0 range of 28–35 dB–Hz for the weak signal, the compensation error can be obtained as shown in Figure 8.

Figure 8 shows that if the signal strength is low, the effect of the C/N0 estimation error becomes rather large. In case the C/N0 is estimated to be low, the open loop gain is increased. As a result, the natural frequency of the close loop increases and noise increases as the bandwidth is increased. On the contrary, if the C/N0 is estimated to be high, the open loop gain is decreased and the response becomes slow down. Therefore, the effect of the C/N0 estimation error on the tracking performance is presented by the simulation in Section 3.

## 3. Simulation and Results

In this section, we evaluated the performance of the proposed algorithm by computer simulations. Simulations are divided into two parts. At the first part, signal overlapping algorithm is verified using Spirent simulator output, and at the second part, the tracking performance of the proposed RLL algorithm is verified under the several values of signal strength using the software-based signal generator output.

### 3.1. Verifying the Signal Overlapping Algorithm

**Figure 9:**
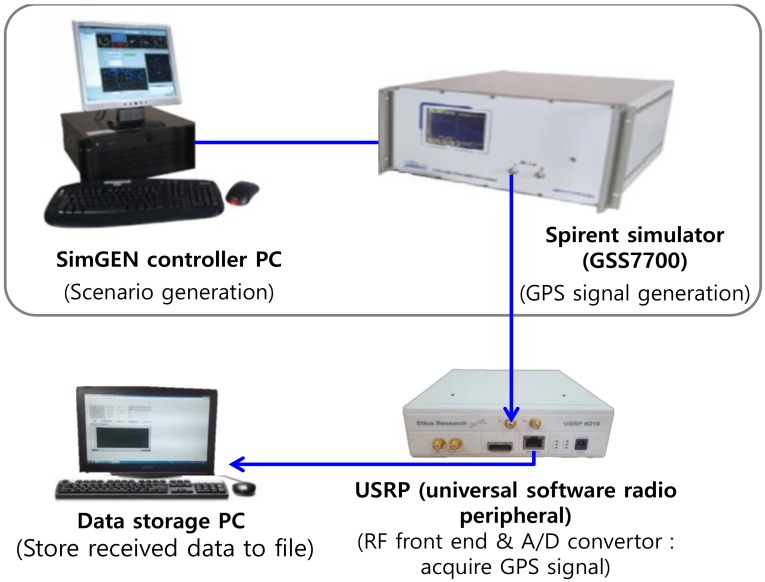
Signal collection using Spirent simulator.

In order to verify the improvement of SNR by the overlapping algorithm, a Spirent GSS7700 GPS simulator (Spirent, Crawley, West Sussex, UK) was used to collect 10 GPS satellites signals for a vehicle rotating at 5 Hz with an antenna which has a beam width of 180 degree as shown in Figure 9. The strength of the received GPS satellites signals was 41.7–50.6 dB–Hz (mean: 46.5 dB–Hz, standard deviation: 3.2 dB–Hz).

The collected signals were stored as a computer file. The navigation data were decoded by processing the stored file with the software-based GPS receiver developed by Chungnam National University, and the satellites’ and vehicle’s positions were obtained from the receiver. Next, the roll angles of the satellites were calculated and the time delay for the rotation frequency of 5 Hz were obtained as the pseudocode of Table 1. Finally, synchronization was performed by delaying and overlapping the correlator outputs of each satellite signals. 

**Figure 10:**
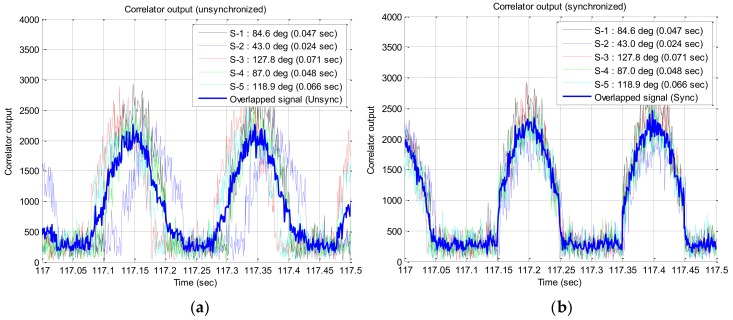
Correlator output: (**a**) before time sync; (**b**) after time sync.

**Figure 11:**
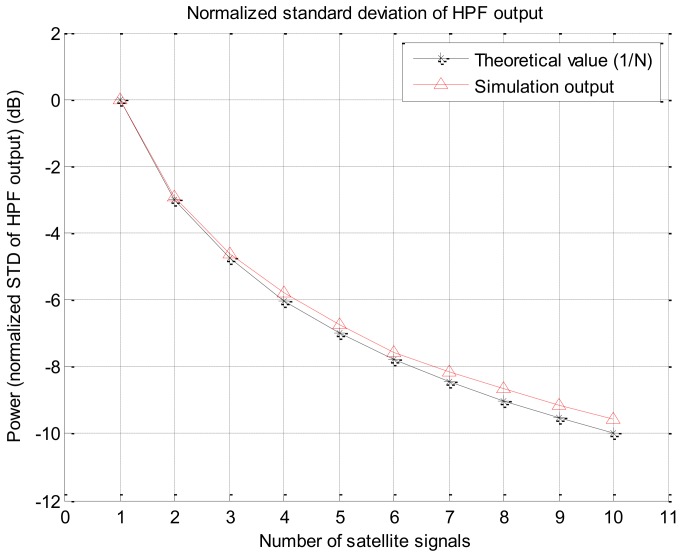
Noise power (as the normalized standard deviation of high pass filter output) w.r.t to the number of overlapped signals.

Correlator outputs before and after time synchronization are shown in Figure 10a,b, in which only the results of five satellite signals among 10 satellite signals are given in order to improve the readability. From Figure 10b, it is shown that the correlator outputs for each satellite signals are well synchronized in time. The noise power of the correlator output with respect to the number of overlapped signals is given in Figure 11. It can be confirmed from the figure that the measured power is similar to the theoretical value that the noise power decreases to 1/N when N signals are overlapped. By the simulation results using Spirent simulator output, it is verified that the noise of the correlator output decreases by signal overlapping algorithm.

**Figure 12:**
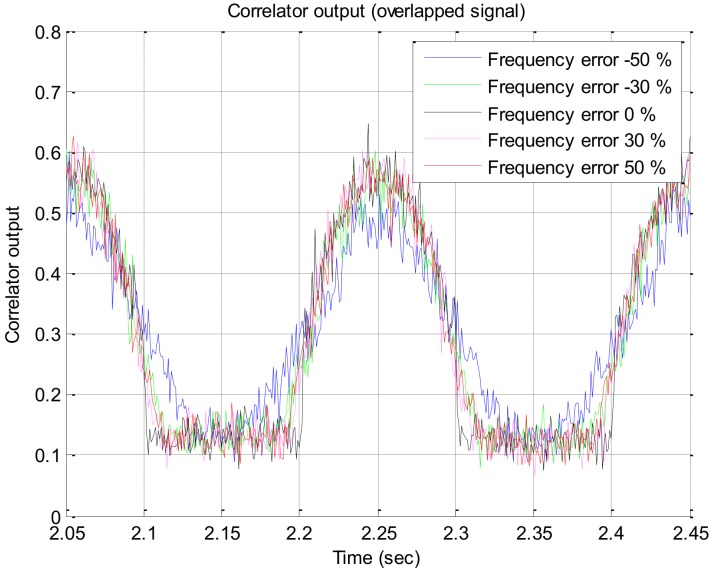
Overlapped signal with different estimation error.

**Figure 13:**
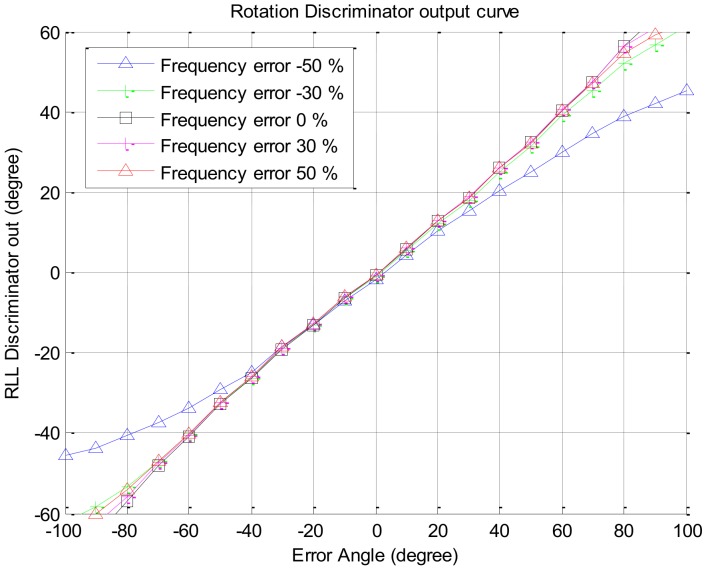
Discriminator output w.r.t the estimation error.

**Figure 14:**
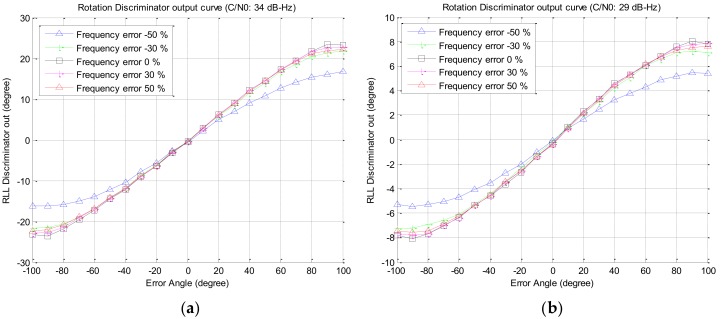
Discriminator output w.r.t the estimation error (using software-based signal generator): (**a**) C/N0 34 dB–Hz; (**b**) C/N0 29 dB–Hz.

Since the estimated rotation frequency of the vehicle is used to calculate the time delay to overlap multiple satellite signals, the effect of the estimation error of the rotation frequency on the overlapped signal is analyzed in this section. Using the same Spirent simulator’s output described above, for the case that error of the estimated frequency is −50–50%, the correlator output of the overlapped signal and the output of the rotation discriminator are presented in Figure 12 and Figure 13, respectively. From Figure 13, it can be seen that when the estimation error of the rotation frequency is −50%, the response of the discriminator is slightly lowered only in the region where the input error angle is larger than 30 degrees, but it has little effect on the output of the discriminator in the other regions. Additional experiments were performed using the output signal of the software-based signal generator for signal strengths of 34 dB–Hz and 29 dB–Hz, and the outputs of the discriminator are presented in Figure 14a,b, respectively, which are similar to the results in Figure 13.

### 3.2. Proposed Enhanced RLL Algorithm

**Figure 15:**
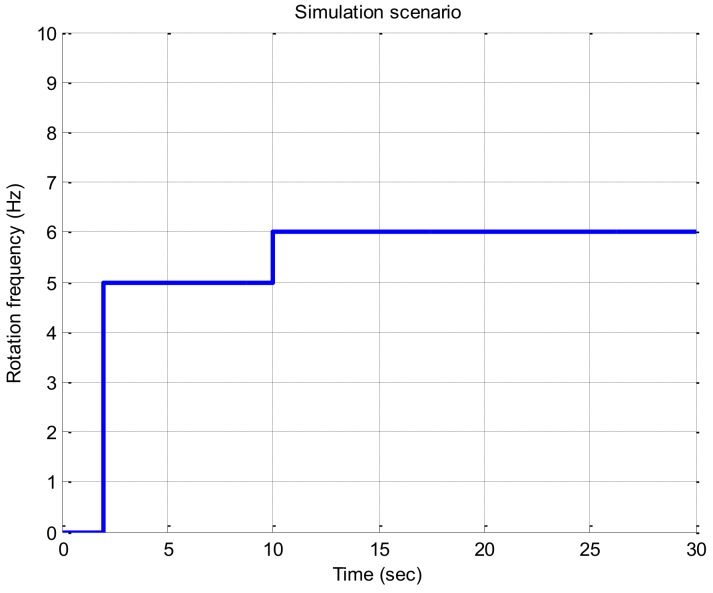
Simulation scenario.

In this section, simulations were performed to evaluate the performance of the proposed algorithm according to the signal strength. In order to change the signal strength and the rotation frequency of the vehicle over time, a satellite signal was generated using a software-based signal generator developed by Chungnam National University and stored as a computer file. In this simulation, the beam width of the antenna was set to 180 degree as in the previous case of using Spirent simulator and 12 GPS satellite signals were simulated. The rotation scenario of the vehicle was set to start from non-rotating state as shown in Figure 15, rotate at 5 Hz after 2 s, and rotate at 6 Hz after 10 s. In this simulation, the rotation frequencies of 5 and 6 Hz are chosen to show the simulation results clearly in figures but the rotation frequency itself does not likely to affect the performance of the proposed algorithm. In order to verify the roll angle estimation performance of the proposed algorithm, in this paper, we used a software receiver to track the code and carrier of the signal stored in the computer file and generate the correlator outputs. In addition, the correlator outputs were synchronized by using the time delay calculated in the same manner as in Section 3.1, and were overlapped on each other, and then the roll angle and the rotation frequency were estimated using the RLL algorithm. In this simulation, the difference from Section 3.1 is that the rotation frequency of the vehicle for calculating the time delay was estimated from the output of RLL instead of using the known true rotation frequency, and the signal strength to compensate the rotation discriminator was applied as the known true value, not the estimated value.

**Figure 16:**
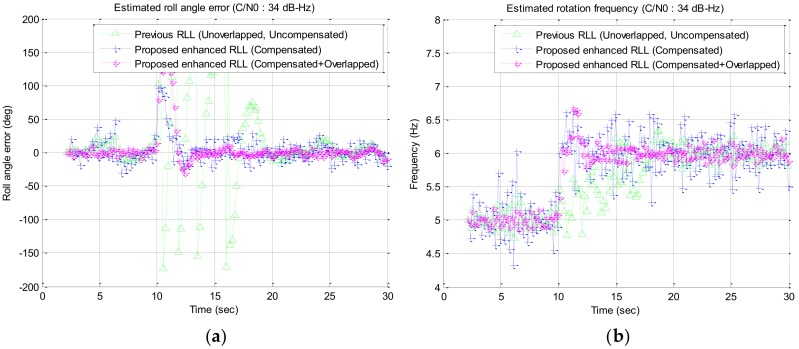
Simulation results for the signal strength of 34 dB–Hz: (**a**) estimated roll angle error; (**b**) estimated rotation frequency.

**Figure 17:**
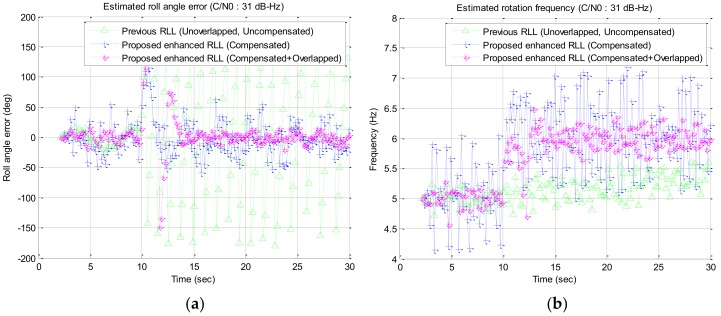
Simulation results for the signal strength of 31 dB–Hz: (**a**) estimated roll angle error; (**b**) estimated rotation frequency.

**Figure 18:**
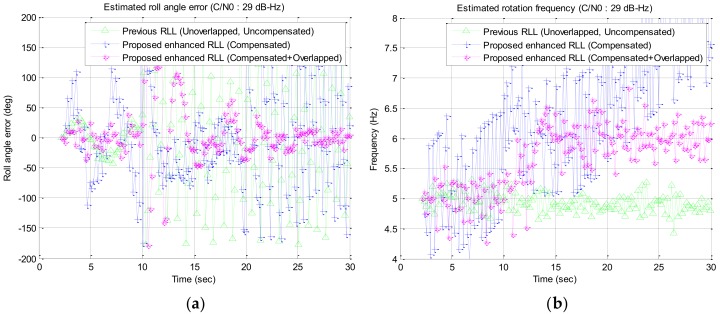
Simulation results for the signal strength of 29 dB–Hz: (**a**) estimated roll angle error; (**b**) estimated rotation frequency.

**Figure 19:**
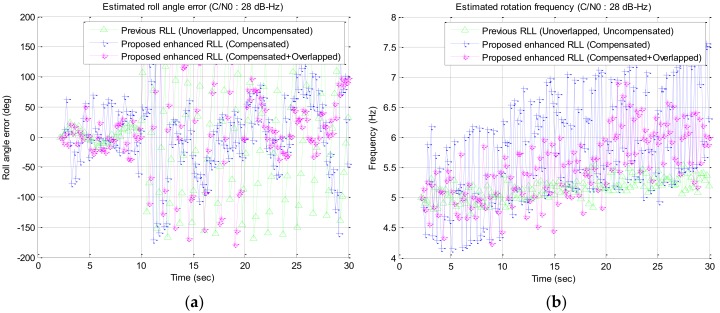
Simulation results for the signal strength of 28 dB–Hz: (**a**) estimated roll angle error; (**b**) estimated rotation frequency.

In the simulation study, a wide range of signal strengths with a step of 1 dB are used as the input signal strength but only interested signal strengths of C/N0 34 dB–Hz to 28 dB–Hz are described in Figure 16, Figure 17, Figure 18 and Figure 19 and Table 2. Among the results, the estimated roll angle error and the estimated rotation frequency for the four signal strengths are graphically presented in Figure 16, Figure 17, Figure 18 and Figure 19. The average and standard deviation of the roll angle estimation error and the rotation frequency for each signal strength, the convergence time of the rotation frequency are presented in Table 1. In the Algorithm column of the table, Proposed RLL-C and Proposed RLL-C/O means that “only Compensation algorithm is used” and “Compensation with Overlapping algorithm with 12 signals is used” respectively. Here, four cases were simulated considering the overlap of the satellite signals and the compensation of the rotation discriminator response for each signal strength, but the results are presented only for the three modes because there was little performance change when only the satellite signals were overlapped without compensating the discriminator response. The determination of the failure of the convergence of RLL algorithm is based on the case that the estimated roll angle error exceeds 180 degrees and does not converge to 0 degree when the rotational frequency changes. When the convergence failure is determined, it can be confirmed from the graphs that estimated rotation frequency does not converge to 6 Hz. Mean and standard deviation in Table 2 were measured after the estimated rotation frequency had converged and these values were the results from one simulation.

When the signal strength was 34 dB–Hz, the estimated roll angle was converged after about 10 s after the rotation frequency was changed in the case of using one satellite signal without compensating the discriminator response (previous RLL). When the discriminator response was compensated (proposed RLL-C), the roll angle was estimated continuously, and the rotation estimation error was reduced when the discriminator response was compensated and 12 signals were overlapped (Proposed RLL-C/O) as shown in Figure 16. In this case, the estimation error became larger in Proposed RLL-C. This is because the open loop gain becomes larger as the discriminator response is compensated, and the noise bandwidth of the closed loop becomes larger in turn. 

In case the signal strength was 31 dB–Hz, the roll angle could not be estimated in Previous RLL. However, the roll angle was estimated continuously in Proposed RLL-C, and the rotation estimation error was reduced in proposed RLL-C/O as shown in Figure 17. 

When the signal strength was 29 dB–Hz, the roll angle could not be estimated in Previous RLL or in Proposed RLL-C. However, the roll angle was estimated after about 5 s after the rotation frequency was changed in Proposed RLL-C/O as shown in Figure 18. 

In case the signal strength was 28 dB–Hz, the roll angle was estimated after about 10 s after the rotation frequency was changed only in Proposed RLL-C/O as shown in Figure 19. However, since the mean and standard deviation of the roll angle error were high, it is considered to be the limit of the signal strength that can be tracked by the proposed algorithm.

In this simulation, the marginal point where RLL stably tracks the roll angle was determined by the roll angle error and the convergence time. In Previous RLL the phase error was high and the convergence time was long at the signal strength of 32 dB–Hz, so 33 dB–Hz was determined as a marginal tracking point. In Proposed RLL-C the roll angle error was small and convergence time was short at the signal strength of 31 dB–Hz, so this strength was considered as a marginal point. In Proposed RLL-C/O the roll angle error was high and the convergence time was long at the signal strength of 28 dB–Hz, so 29 dB–Hz was considered as a marginal point. Overall, by using the proposed enhanced RLL algorithm, the marginal point for stable signal tracking is improved by 4 dB from 33 dB–Hz to 29 dB–Hz.

The improvement by signal overlapping algorithm is theoretically 10.8 dB ($10\log_{10}12$). Actually, however, the performance improvement was about 4 dB because the noise characteristic was degraded by increasing the response of the rotation discriminator. When the signal strength was 29 dB–Hz, an additional gain of 8.3 was applied for the discriminator compensation as shown in Figure 7, and the noise power increased about 7.7 dB as is described below due to the additional gain. Therefore, the performance improvement of 3.1 dB was expected.

The noise bandwidth ($B_{n}$) and the 2nd order RLL tracking loop is shown as follows [10]:

(5) $$B_{n}\;=\;\frac{\omega_{n}}{8\zeta }\left(4\zeta^{2}+1\right),$$

where, $\omega_{n}$ and ζ denote the natural frequency and the damping ratio. If additional gain due to the discriminator compensation is d, then ζ and $\omega_{n}$ become $\sqrt{d}\zeta$ and $\sqrt{d}\omega_{n}$ respectively, and thus the modified noise bandwidth (${B^{\prime }}_{n})$ is given as follows:

(6) $$B_{n}^{\prime }\;=\;\frac{\omega_{n}}{8\zeta }\left(4{d\zeta }^{2}+1\right)\;.$$

Therefore, the modified noise bandwidth is $5.87B_{n}$, which means that the noise power increased by 7.7 dB ($10\log_{10}5.87$).

Through the simulation, the necessity for the compensation of the discriminator response was shown and it was verified that the roll angle estimation error was reduced and the roll angle could be estimated even at the lower signal strength by overlapping multiple signals.

### 3.3. Effect of the C/N0 Estimation Error

**Figure 20:**
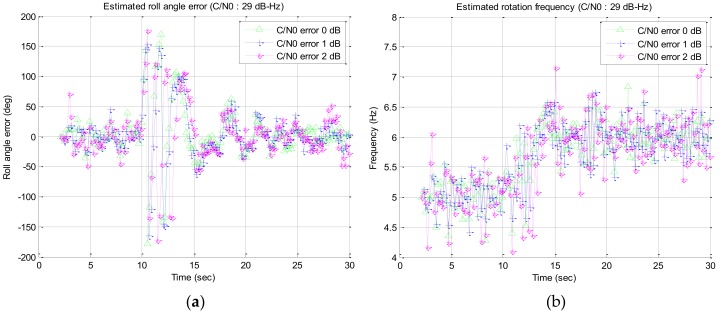
Simulation results for the signal strength of 29 dB–Hz with C/N0 estimation error: (**a**) estimated roll angle error; (**b**) estimated rotation frequency.

In this section, the effect of the C/N0 estimation error on the tracking performance of roll angle and rotation frequency is simulated. The effect of the rotation frequency error was not analyzed because the overlapping algorithm used the RLL algorithm output as the estimated rotation frequency in Section 3.2. Therefore, the simulations only for the effect of the C/N0 estimation error were performed in this section. The simulation was performed for the case that the signal strength is 29 dB–Hz as C/N0 with proposed enhanced RLL (compensated + overlapped) which is the marginal point as described in Section 3.2. The C/N0 error was assumed to be 1 dB–Hz and 2 dB–Hz with Gaussian random distribution in reference to the Pini et al. [11]. The simulation results are shown in Figure 20 and mean and standard deviation of the tracking error are summarized in Table 3. In this table, None, 1 dB–Hz, and 2 dB–Hz in C/N0 error column mean that no C/N0 error was applied, 1 dB–Hz and 2 dB–Hz of C/N0 error was applied, respectively.

In Table 3, the mean and standard deviation for the case 1 were the result from one simulation, and those for the case 2 and 3 were the averaged values from ten simulations with Gaussian random error. Through these simulation results, it is shown that the C/N0 estimation error increases the standard deviation of the tracking error, but the estimated roll angle and rotation frequency are still converged to the true value.

## 4. Conclusions

In this paper, in order to estimate the roll angle of a rotating vehicle using received GPS signals in a weak signal environment, an enhanced RLL algorithm is proposed. This algorithm is a method to increase the SNR of correlator output by synchronizing and overlapping multiple GPS signals received at a time and the performance is verified using GPS signals generated by a commercial Spirent simulator. In addition, it was found that the response of an RLL rotation discriminator is decreased under the weak signal environment. Therefore, by compensating for the decreased response the estimation performance of the roll angle at transient region where the rotation frequency changes are improved. However, the compensation of the decreased response also increases the noise bandwidth and as a result the overall noise is increased too.

In conclusion, it is verified by the computer simulation that the roll angle can be estimated at 4 dB lower signal down to 29 dB–Hz of C/N0 when 12 satellite signals are used with proposed algorithm in compared to the conventional RLL method. In this low-signal environment, even though the standard deviation of the roll angle estimation error becomes rather as high as 20.4 degrees, the proposed algorithm successfully estimates the roll angle. In addition, the effect of the C/N0 estimation error was analyzed, and it is verified that the roll angle is still normally estimated under the C/N0 estimation error of 2 dB–Hz. Moreover, the compensation algorithm for the RLL rotation discriminator in weak signals can be applied in the same way in phase lock loop or in delay lock loop to compensate for the discriminator response where the structure of the discriminator is similar to RLL.

## Figures and Tables

**Table 1:** Pseudo code to calculate time delay to synchronize multiple correlator output signals.

C_eg = [ −sin(lat) × cos(lon); −sin(lat) × sin(lon); cos(lat);		// transformation matrix
−sin(lon); cos(lon); 0;		// lat: latitude of a vehicle
−cos(lat) × cos(lon); −cos(lat) × sin(lon); −sin(lat) ];		// lon: longitude of a vehicle
R = S − P;	// S, P: satellite’s and vehicle’s position in ECEF	
u_e = R/norm(R);	// unit vector to the direction of the satellite	
u_g = C_eg × u_e;	// u_e represented by NED	
Az = atan(u_g(2)/u_g(1));	// Azimuth of the satellite	
El = atan(u_g(3)/norm(u_g(1), u_g(2));	// Elevation of the satellite	
if (sin(Az − phi_p) > 0)	// phi_p: heading of the vehicle	
phi_s = (pi − El) × sin(Az − phi_p);	// roll angle of the satellite	
else	// pitch angle is assumed to be zero	
phi_s = El × sin(Az − phi_p);	// roll angle of the satellite	
end if		
t_d = phi_s/(2 × pi × f);	// t_d: time delay to synchronize the signals	

ECEF: Earth-Centered, Earth-Fixed; NED: North-East-Down.

**Table 2:** Summarized simulation results.

Simulation Conditions		Simulation Results				
Signal Strength (C/N0)	Algorithm	Estimated Roll Angle Error (Degree)		Estimated Rotation Frequency (Hz)		Converged Region
		Mean	STD	Mean	STD	
34 dB–Hz	Previous RLL	0.9	8.3	6.00	0.12	20–30 s
	Proposed RLL-C	1.7	12.0	6.01	0.29	12–30 s
	Proposed RLL-C/O	0.0	4.5	6.00	0.08	13–30 s
33 dB–Hz	**Previous RLL**	**2.16**	**8.2**	**6.01**	**0.15**	**25–30 s**
	Proposed RLL-C	−5.25	20.5	6.05	0.53	14–30 s
	Proposed RLL-C/O	−0.59	5.1	6.00	0.10	14–30 s
32 dB–Hz	Previous RLL	20.0	18.1	5.98	0.14	27–30 s
	Proposed RLL-C	−4.05	21.9	6.06	0.59	13–30 s
	Proposed RLL-C/O	−0.36	10.4	6.00	0.12	13–30 s
31 dB–Hz	Previous RLL	-				(Fail)
	**Proposed RLL-C**	**−4.6**	**25.0**	**6.06**	**0.60**	**14–30 s**
	Proposed RLL-C/O	−0.1	7.6	6.00	0.16	14–30 s
30 dB–Hz	Previous RLL					(Fail)
	Proposed RLL-C					(Fail)
	Proposed RLL-C/O	−5.4	12.9	6.00	0.21	15–30 s
29 dB–Hz	Previous RLL					(Fail)
	Proposed RLL-C					(Fail)
	**Proposed RLL-C/O**	**−2.0**	**20.4**	**6.01**	**0.27**	**15–30 s**
28 dB–Hz	Previous RLL					(Fail)
	Proposed RLL-C					(Fail)
	Proposed RLL-C/O	28.2	38.5	6.01	0.40	20–30 s
Bolded lines denote the marginal points where RLL stably track the roll angle. – Previous RLL (algorithm is not applied): 33 dB–Hz – Proposed RLL-C (compensation algorithm is applied): 31 dB–Hz – Proposed RLL-C/O (compensation and overlapping algorithms are applied): 29 dB–Hz						

**Table 3:** Simulation results with estimated C/N0 error (C/N0: 29 dB–Hz).

Test Case	C/N0 Error	Simulation Results						
		Estimated Roll Angle Error (Degree)			Estimated Rotation Frequency (Hz)			Converged Region
		Mean	STD	Increment	Mean	STD	Increment	
Case 1	None	−2.0	20.4	-	6.01	0.27	-	15–30 s
Case 2	1 dB–Hz	−2.0	20.8	2.0 %	6.01	0.33	22.2 %	15–30 s
Case 3	2 dB–Hz	−2.0	21.8	6.9 %	6.02	0.38	40.7 %	15–30 s
